# A new *Dermatohomoeus* from the Ulu Temburong forest, Brunei (Coleoptera, Leiodidae)

**DOI:** 10.3897/BDJ.14.e202669

**Published:** 2026-07-20

**Authors:** Immanuel John S. Lasco, Nick Rossen, Riccardo Focaia, Niccolò Rossi, Bram van Rijn, Hugo Koopmans, Peter Koomen, David Alford, Boukje Beuger, Dumitru Diaconu, Francesco Fiorentino, Rizmoon Nurul Zulkarnaen, Larry Holbrook, Paula A. Gómez-Zapata, Rafhiah Siti Kahar, Massimo Delledonne, Daniele Cicuzza, Iva Njunjić, Menno Schilthuizen

**Affiliations:** 1 Universiti Brunei Darussalam, Bandar Seri Begawan, Brunei Universiti Brunei Darussalam Bandar Seri Begawan Brunei; 2 HAN University of Applied Science, Nijmegen, Netherlands HAN University of Applied Science Nijmegen Netherlands; 3 Taxon Expeditions, Leiden, Netherlands Taxon Expeditions Leiden Netherlands; 4 University of Verona, Verona, Italy University of Verona Verona Italy; 5 Museum National d'Histoire Naturelle, Paris, France Museum National d'Histoire Naturelle Paris France; 6 Naturalis Biodiversity Center, Leiden, Netherlands Naturalis Biodiversity Center Leiden Netherlands; 7 Taxon Expeditions B.V., Leiden, Netherlands Taxon Expeditions B.V. Leiden Netherlands; 8 Leiden University, Leiden, Netherlands Leiden University Leiden Netherlands; 9 Taxon Foundation, Leiden, Netherlands Taxon Foundation Leiden Netherlands

**Keywords:** Borneo, Brunei, citizen science, Coleoptera, Leiodidae, new species, taxonomy

## Abstract

**Background:**

‘Taxon expeditions’ are field courses/expeditions for mixed teams of scientists, students and lay people with the aim to do biodiversity inventories, particularly of cryptobiota and to discover and describe new species.

**New information:**

During a taxon expedition to the Kuala Belalong Field Studies Centre and surrounding area of the Ulu Temburong Forest in Brunei, leaf litter sampling yielded a specimen of a previously unknown species of the round fungus beetle genus *Dermatohomoeus* Hlisnikovský (Leiodidae, Leiodinae, Pseudoliodini). Using the limited time and facilities available, a scientific description was prepared by a number of the expedition participants. The new species is described, pictured and compared with related species from South and Southeast Asia.

## Introduction

Several genera of round fungus beetles (Leiodidae) are common and speciose inhabitants of the leaf litter layer of lowland tropical rainforests of Southeast Asia. In Borneo, notable representatives are *Colenisia* Fauvel and *Dermatohomoeus* (Leiodidae, Leiodinae) and *Ptomaphaminus* Perreau and *Ptomaphaginus* Portevin (Leiodidae, Cholevinae) (Schilthuizen et al. 2017, Schilthuizen et al. 2018, Švec 2022, Hu et al. 2024). In addition, many other leiodid genera are present in this habitat, though usually in smaller numbers of species and at lower abundances. As these beetles are generally small (1-3 mm in length), cryptic (they occur hidden in the leaf litter layer and require special collecting techniques to obtain them in numbers) and uniform (most species can only be confidently identified by their genitalia), they remain understudied. The genus *Dermatohomoeus* presently contains 56 species distributed in a wide geographic area extending over the whole of oriental Asia (from India to Japan) and Oceania. Only four species have been reported from Borneo: *D. hamatus* Švec 1998 from Kalimantan, *D. maliauensis* Schilthuizen, Otani & Seip, 2017 from Sabah, *D. portevini* (Champion 1923) from Sarawak (but recorded also from India, Japan, Nepal, Thailand and Vietnam) and *D. sarawaki* Hlisnikovský 1965, also from Sarawak (Schilthuizen et al. 2017, Švec 2022). Given the unpopularity of this type of microcoleoptera, the size of the island and the variety of its natural habitats, it is quite likely that the true diversity in this genus in Borneo is much greater. In this paper, we describe a new species that was found during a taxon expedition (see below) in the lowland primary rainforest of Brunei. The new species here described is the first species from Brunei.

## Materials and methods

### Fieldwork

**Figure 1.**
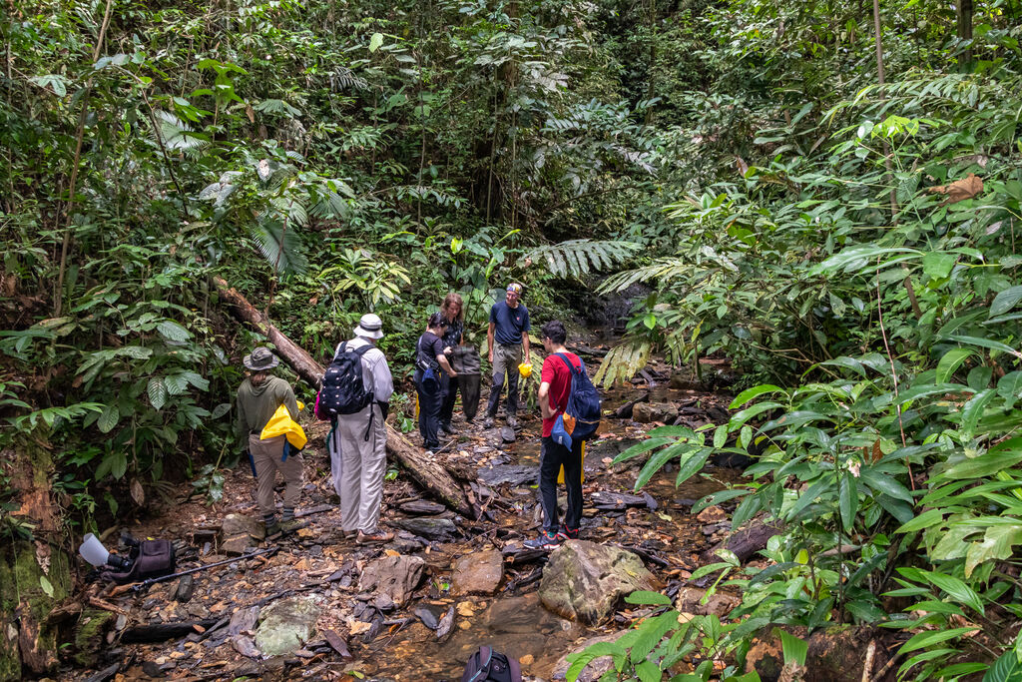
Leaf litter sieving on a taxon expedition at Kuala Belalong Field Studies Centre (photo: René Krekels).

The sampling site was located at the Ashton Trail (4.545°N, 115.157°E) adjacent to the Kuala Belalong Field Studies Centre (KBFSC), located in the Ulu Temburong National Park, Temburong District, Brunei Darussalam, in primary lowland dipterocarp rainforest (Fig. 1). Sampling was performed in the afternoon of 8 October 2025, roughly between 16:00 and 17:00 hours. Leaf litter was collected manually and sieved on site using standard leaf litter sieves with a 1 cm^2^ mesh size (manufactured by BioForm, Germany). The flow-through was placed in fabric bags and suspended within two Winkler collectors (manufactured by Entowinkler, Austria) back at the KBFSC. Collection containers filled with 70% ethanol were examined daily for the duration of 7 days.

### Molecular work

Tissue samples underwent genetic analysis in a portable field laboratory set up in the field centre. Genomic DNA extraction was performed on soft tissue from the abdomen of the specimen using the *DNeasy Blood & Tissue Kit* (Qiagen, Hilden, Germany) following a modified protocol (Menegon et al. 2017, Maestri et al. 2019). Samples were incubated in ATL buffer with proteinase K at 56°C for at least 3 h, followed by overnight incubation at room temperature. DNA was eluted twice in 50 µl of TE buffer to maximise yield. The integrity of the extracted DNA was assessed by electrophoresis on a 0.8% agarose gel and the concentration was estimated by comparison with mass ladders of known DNA quantities.

The mitochondrial cytochrome *c* oxidase subunit I (COI) barcoding region was amplified using the universal "Folmer" primers (LCO1490 and HCO2198) (Folmer et al. 1994), each tailed at the 5' end with Oxford Nanopore adapter sequences: LCO1490: 5'–(TTTCTGTTGGTGCTGATATTGC)GGTCAACAAATCATAAAGATATTGG–3' and HCO2198: 5'–(ACTTGCCTGTCGCTCTATCTTC)TAAACTTCAGGGTGACCAAAAAATCA–3'. PCR products were verified on a 0.8% agarose gel to confirm the expected amplicon size (~ 710 bp) and absence of non-specific bands or contamination. Successful amplicons were uniquely indexed using the PCR Barcoding Kit (Oxford Nanopore Technologies, Oxford, UK). Indexed amplicons were pooled in equimolar concentrations and purified using 0.5× AMPure XP magnetic beads (Beckman Coulter, Brea, CA, USA). The pooled library was quantified with a Qubit 4 fluorometer (Thermo Fisher Scientific, Waltham, MA, USA).

Sequencing libraries were prepared from 1 µg of pooled amplicons using the Ligation Sequencing Kit V.14 (SQK-LSK114; Oxford Nanopore Technologies) according to the manufacturer’s instructions, omitting the fragmentation and end-prep steps. Sequencing was performed on a MinION device (Oxford Nanopore Technologies) using an R10.4.1 flow cell. Data acquisition and run control were managed with MinKNOW v.25.05.14, with a target output of 5,000 reads.

### Bioinformatic analysis

POD5 files were basecalled and demultiplexed with Dorado v.1.1.0 (*super-accurate* model) with the dual-barcode option (*--barcode-both-ends*) enabled. The resulting FASTQ files were processed with ONTrack2 (Maestri et al. 2019), which clusters reads and generates a consensus sequence from the most abundant cluster to minimise potential errors. Only sequences with a minimum quality score of 20 and a length between 705 and 715 bp were retained and read clustering was performed at 90% sequence identity. Consensus sequence was queried against a locally downloaded NCBI COI database (NCBI nucleotide database release 15 September 2025). The sequence of the holotype was uploaded to Barcode of Life Database (BOLD; www.boldsystems.org) under accession number: TXEX082-26.

### Morphology

Morphological study of the specimen was done in the KBFSC field laboratory, using the Taxon Expeditions equipment. We used a Nikon SMZ445 microscope fitted with 10× eye pieces to examine morphological features to a maximum magnification of 35×. Photographs were taken either with an iPhone through the eyepiece of the microscope or with an OM System OM1-II fitted with a 90 mm macro lens (OM System) and a 2× teleconverter MC-20 of Olympus and three Ulanzi VIJIM VL120 RGB LED video lights with silicone light diffusers. Focus stacking was done with Helicon Focus v.8.3.9 (Kharkiv, Ukraine) using method C. Reproductive structures were dissected and embedded on a microscope slide in polyvinylpyrrolidone (Lompe 1986). Images of the aedeagus were traced by hand and interpreted in the style of Daffner (1988). The measurements were taken using ImageJ (ver. 1.54G, 64-bit, Windows 11). Measurements of body parts were calibrated with an image of a ruler taken at the same magnification as the specimen. The holotype was deposited in the Zoology Museum of Universiti Brunei Darussalam.

## Taxon treatments

### Dermatohomoeus sagittarius

Lasco, Rossen & Schilthuizen sp. nov.

A71B905D-C181-5FE0-9DBA-5F0DA2BE8D26

#### Materials

- **Type status:** Holotype. **Occurrence:** individualCount: 1; sex: male; lifeStage: adult; otherCatalogNumbers: UBDM.3.07895; occurrenceID: 352CE81E-6723-5706-B2D5-FA55EDB0B621; **Taxon:** scientificName: Dermatohomoeus sagittarius Lasco et al., 2026; kingdom: Animalia; phylum: Arthropoda; class: Insecta; order: Coleoptera; family: Leiodidae; genus: Dermatohomoeus; specificEpithet: sagittarius; taxonRank: species; scientificNameAuthorship: Lasco et al.; nomenclaturalCode: ICZN; **Location:** higherGeography: Borneo; Brunei Darussalam; Temburong district; Kuala Belalong Field Studies Centre; continent: Asia; island: Borneo; country: Brunei; stateProvince: Temburong; locality: Kuala Belalong Field Studies Centre; verbatimElevation: 150 m; decimalLatitude: 4.54; decimalLongitude: 115.17; **Identification:** identifiedBy: Menno Schilthuizen; dateIdentified: 14-10-2025; **Event:** samplingProtocol: Leaf litter sampling; samplingEffort: 10 l of leaf litter; eventDate: 08-10-2025; year: 2025; month: 10; day: 8; habitat: Primary dipterocarp rainforest; **Record Level:** type: PhysicalObject; institutionID: UBD; collectionID: UBDM; institutionCode: UBDM; ownerInstitutionCode: UBDM; basisOfRecord: PreservedSpecimen; **Material Entity:** preparations: aedeagus, dry mounted; disposition: in collection of the Zoology Museum, Universiti Brunei Darussalam

#### Description

**Figure 2.**
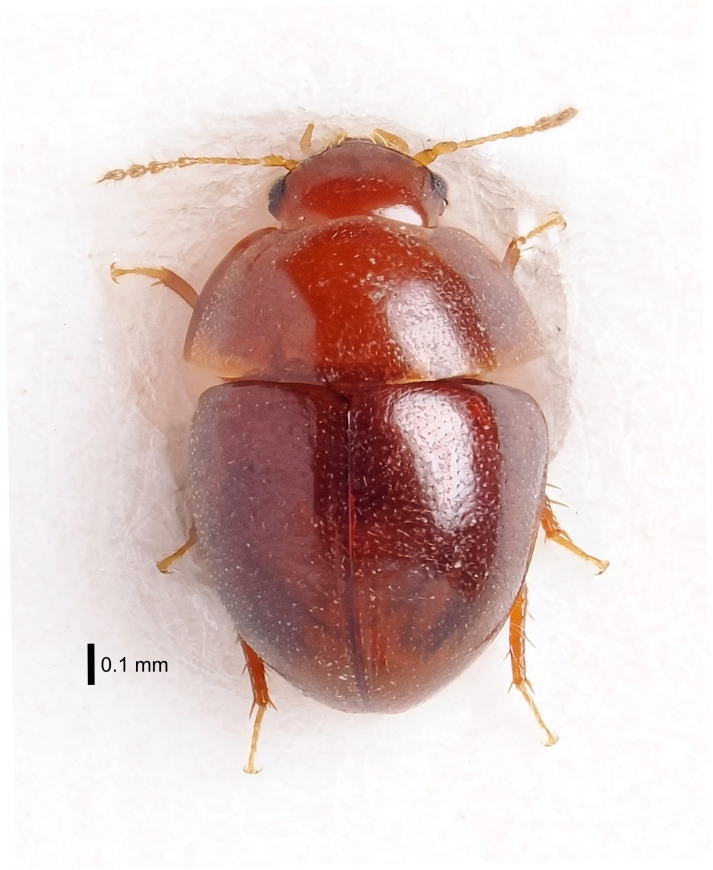
*Dermatohomoeus sagittarius* sp. nov., habitus, dorsal view.

Habitus (Fig. 2). Total body length 1.37 mm. Shape of body ovoid (Fig. 2), pronotum barely wider than the elytra at the shoulders. Dorsum amber-coloured. Legs, mouth parts and antennomeres translucent yellowish- to amber-coloured. The underside is amber-coloured. The coxae are dark brown.

**Head**. Well-developed subglobose eyes that are as wide as the first antennomere. Very distinct strong and dense puncturation on dorsal surface of the head separated by spaces that are as wide as the punctures themselves. Several punctures with short laterally directed recumbent setae. The antennae are clavate and covered with erect setae, densest and longest at the two basal and last three terminal segments. All antennomeres longer than wide, the 8^th^, 9^th^ and 10^th^ only marginally so.

**Pronotum**. Broadest at the base, which is straight and very weakly emarginated before the rectangular posterior angles. Evenly curved sides from base to anterior angles in dorsal view. The base of the pronotum is straight. Pronotal puncturation dense, strong, distinct, punctures drop-shaped with tip orientated anteriorly, separated by interspaces that are, in longitudinal direction, as wide as the punctures themselves and in transverse direction, double the diameter of the punctures. Most punctures with short recumbent setae. Setae predominantly orientated posteriorly. Pronotum with several erect setae along lateral margins. Scutellum smooth.

Elytra broadest at the shoulders, roundly curved to apex. Elytral surface uniformly punctured and setose. Punctures separated predominantly by interspaces that are twice as wide as the punctures themselves, irregularly arranged, equipped with recumbent, posteriorly orientated setae, along lateral margins with several erect setae. The majority of elytral punctures are connected by transverse or oblique strigosity. Sutural stria extending in rostral direction approximately to basal third of elytra. Discal setae are arranged into an irregular grid-like pattern, separated by approximately 0.020-0.035 mm.

**Legs**. Anterior tarsomere I distinctly dilated and elongate. Ratio of length of tarsomere I: tarsomeres II-V (without claws) of anterior tarsus : 0.4.

**Figure 3.**
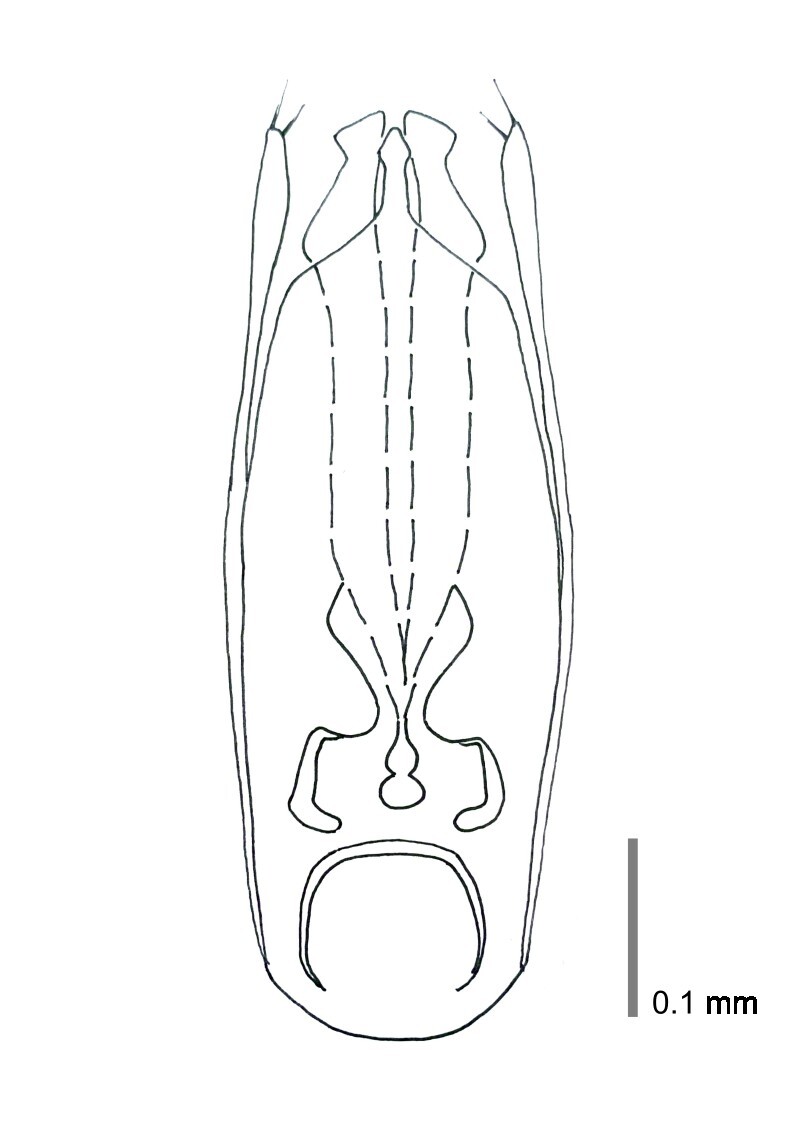
*Dermatohomoeus sagittarius* sp. nov., holotype: aedeagus, dorsal view.

**Figure 4.**
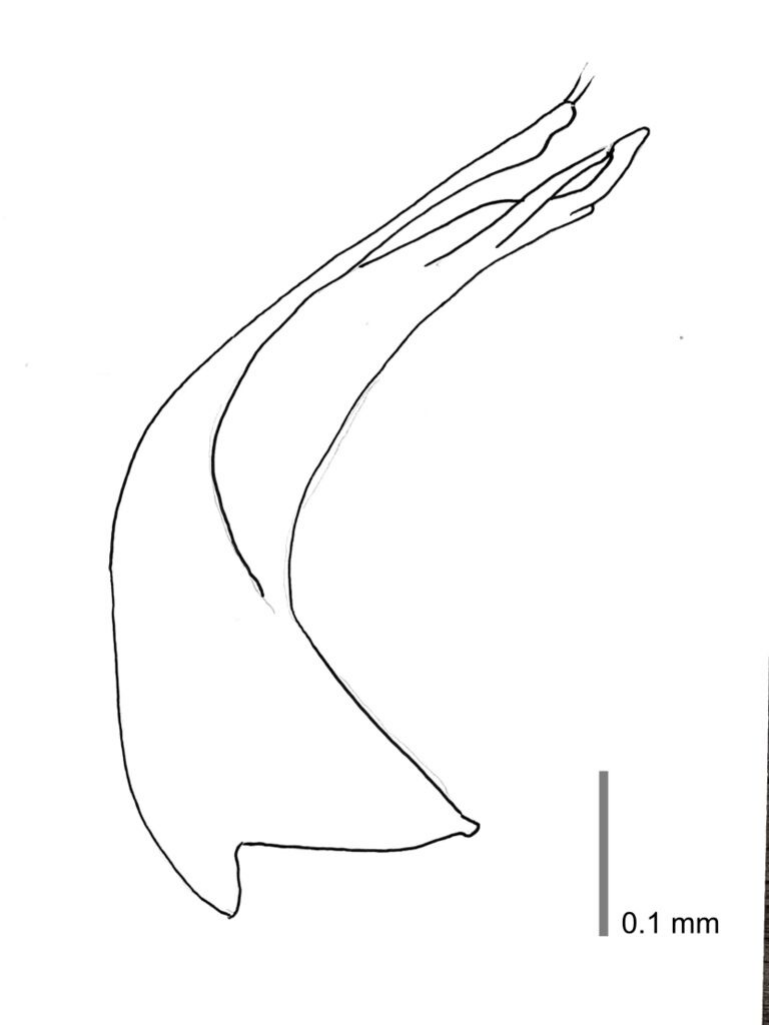
*Dermatohomoeus sagittarius* sp. nov., holotype: aedeagus, lateral view.

Aedeagus in lateral view (Fig. 4) strongly curved at the base, more elongate at the apex, the tip slightly curved upwards. In dorsal view, median lobe apically extended into a narrow, arrow-shaped tip, on either side of which the lateral branches of operculum protrude as spatulae (Fig. 3). The endophallus contains a symmetrical arrangement of two comma-shaped basal sclerites. The parameres do not extend beyond the lateral branches of operculum.

#### Diagnosis

The species of the genus *Dermatohomoeus* are generally very uniform in their appearance, especially those that are of similar dorsal colour and body size. *Dermatohomoeus sagittarius* sp. nov. differs from all known Asian species in the shape of the sclerites of the endophallus, which are comma-shaped, basal and symmetric. The tegmen has a backward orientated tooth on each side before the apex (Fig. 3), a feature shared with *D. rufus* Daffner, 1988 from India.

##### DNA barcode (holotype)

BOLD accession number: TXEX082-26. In total, 3,346 high-quality reads were generated after applying stringent quality and length filters (**Materials and Methods**). Following clustering at 90% sequence identity, the largest cluster comprised 3,047 reads (91%), indicating minimal sample contamination. BLAST searches identified multiple matches to species belonging to the family Leiodidae, with 100% query coverage, but < 89% sequence identity:

5'AAACAAATGTTGGTAAAGAATTGGATCTCCACCTCCTGCAGGGTCAAAAAAGGAAGTATTTAGATTTCGATCTGTTAATAGTATAGTAATAGCACCAGCTAAAACTGGTAAAGATAAAAGCAATAAAAGAGCTGTAATAGCAACTGCTCATACAAATAAAGGTATTTTATCAAAAGTTATACCAGCTCTTCGTATATTAATAACTGTAGTAATAAAATTTACAGCTCCTAAAATAGAGGAGATACCAGCTAGATGTAGTCTAAAAATAGCTAAATCAACTGAAGATCCTCTATGGGCAATATTTGCAGATAGGGGAGGATAAACAGTTCATCCTGTACCTGCTCCATTTTCAACAATTCTTCTTATTAAAAGAAGTGTTAAGGAAGGGGGAAGTAATCAAAATCTTATATTATTTATTCGAGGAAATGCTATATCTGGGGCTCCAAGTATTAAAGGAACCAATCAATTACCAAAGCCTCCAATTATGATTGGCATAACTATAAAAAAAATTATAATAAAAGCATGAGCAGTAACAATTACATTATAAATTTGGTCATCTCCAATTAATCTTCCTGGAGTCCCTAATTCTGCTCGAATCAAAATTCTTAAAGAAGTTCCAACTATACCAGCTCAAGCACCAAAAATAAAGTATAAAGT3'.

#### Etymology

The species epithet (*sagittarius* = archer) refers to the arrow-shaped tip of the median lobe.

#### Taxon discussion

Although the shape of the aegeagus (Fig. 3) in *D. sagittarius* is diagnostic, several other Asian species have a somewhat similar overall structure. Here, we articulate the salient differences.

*D. balkei* Švec, 2009, *D. fulvus* Daffner, 1986, *D. guineensis* Hlisnikovský, 1963 and *D. kaszabi* Hlisnikovský, 1963 from New Guinea and *D. longicornis* Daffner, 1988, *D. obscuratus* Daffner, 1988 and *D. rufus* Daffner, 1988 from India and *D. punctatus* Daffner, 1988 from Thailand all have a median lobe with a more rectangular, spear-shaped point and more rectangular at the base. In *D. balkei*, the ligulae are less round on the outside and lack the 90° angles facing towards each other. In *D. fulvus*, the ligulae do not touch each other in the middle and are slightly bottle-shaped with inclinations at about 2/3 of the length. In *D. guineensis*, the ligulae are more bottle-shaped with inclinations at about 2/3 of the length. In *D. kaszabi*, the ligulae are more parallel to each other, instead of facing more outwards and are more rectangular and bottle-shaped with inclinations at about 2/3 of the length. In *D. longicornis* and *D. obscuratus*, the ligulae lack the hook-like shapes on the outer sides and are more rectangular and bottle-shaped with inclinations at about 2/3 of the length. In *D. rufus*, the ligulae are much longer, surpassing the parameres.

## Discussion

In this paper, we describe a new species of the genus *Dermatohomoeus*, a genus of minute leaf litter dwelling Leiodidae that, in our experience, is both numerous and speciose in the Southeast Asian rainforest. We therefore expect that many more undescribed species await description. The description was prepared by expert-guided, project-based work by untrained citizen scientists, again demonstrating that study of biodiversity by guided non-experts is both possible, productive and important for spreading appreciation for taxonomy and the great numbers of yet unknown species.

## Supplementary Material

XML Treatment for Dermatohomoeus sagittarius
